# Molecular Dynamics Simulations of the Mammalian Glutamate Transporter EAAT3

**DOI:** 10.1371/journal.pone.0092089

**Published:** 2014-03-18

**Authors:** Germano Heinzelmann, Serdar Kuyucak

**Affiliations:** School of Physics, University of Sydney, NSW, Australia; University of Bologna & Italian Institute of Technology, Italy

## Abstract

Excitatory amino acid transporters (EAATs) are membrane proteins that enable sodium-coupled uptake of glutamate and other amino acids into neurons. Crystal structures of the archaeal homolog Glt_Ph_ have been recently determined both in the inward- and outward-facing conformations. Here we construct homology models for the mammalian glutamate transporter EAAT3 in both conformations and perform molecular dynamics simulations to investigate its similarities and differences from Glt_Ph_. In particular, we study the coordination of the different ligands, the gating mechanism and the location of the proton and potassium binding sites in EAAT3. We show that the protonation of the E374 residue is essential for binding of glutamate to EAAT3, otherwise glutamate becomes unstable in the binding site. The gating mechanism in the inward-facing state of EAAT3 is found to be different from that of Glt_Ph_, which is traced to the relocation of an arginine residue from the HP1 segment in Glt_Ph_ to the TM8 segment in EAAT3. Finally, we perform free energy calculations to locate the potassium binding site in EAAT3, and find a high-affinity site that overlaps with the Na1 and Na3 sites in Glt_Ph_.

## Introduction

**Figure 1:**
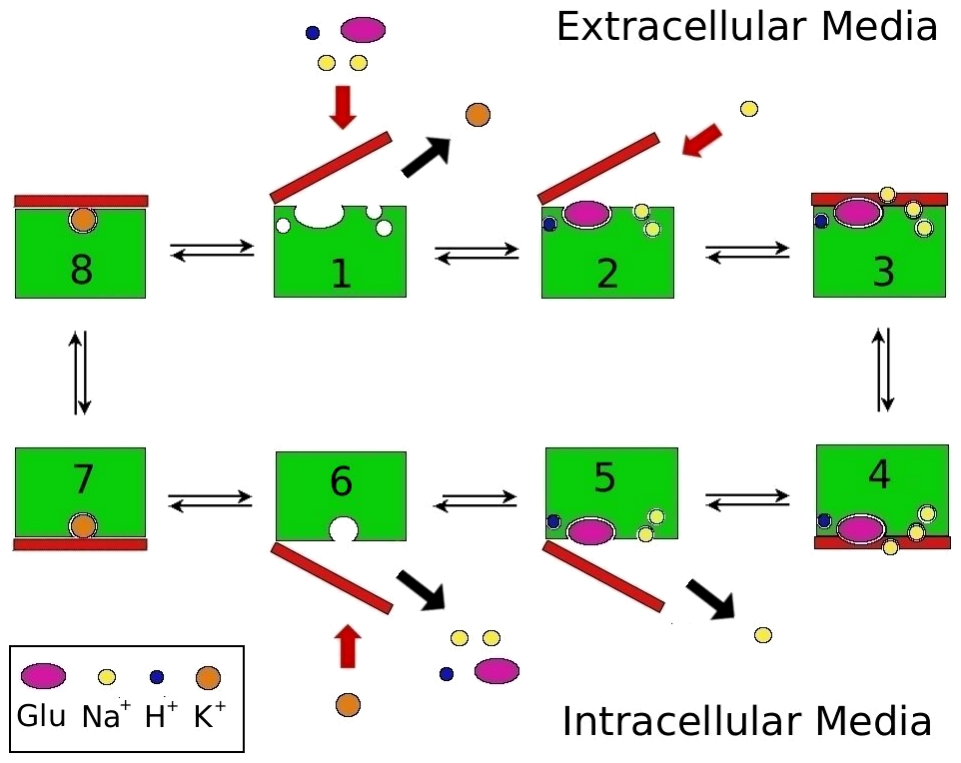
Cartoon showing the functional mechanism of the EAAT transporters. Steps 3–4 denote the translocation of the binding site across the membrane with 3 Na^+^, H^+^, and glutamate bound to EAAT3, while steps 7–8 denote the same with only K^+^ bound. Step 2 shows the binding of the Na2 ion, which binds after the substrate and the closure of the HP2 gate. Step 5 shows the opposite happening in the intracellular media, assuming that the binding and unbinding of ligands are symmetrical.

Excitatory Amino Acid Transporters (EAATs) are responsible for clearing the excess glutamate released at the nerve synapses. There are five subtypes identified in humans, called EAAT1 to EAAT5. Malfunctioning of these proteins has been implicated in many pathological conditions including cerebral ischemia, amyotrophic lateral sclerosis and Alzheimer's disease [1]. The transport mechanism of EAATs involves two half cycles, which enables transport of Glu or Asp against their concentration gradients. The first part involves the binding and translocation of three Na^+^ and one H^+^ ions with the substrate across the membrane while the second half cycle brings the transporter back to its initial conformation by binding and counter-transporting one K^+^ ion [2], [3] (Figure 1). Many mutagenesis and functional experiments have been performed on EAATs in the past, especially in the rodent homologs of EAAT2 and EAAT3, also known as GLT-1 and EAAC1, respectively. We have summarized the results of several studies concerning the residues close to the binding site in Table 1, pointing out the effects of different mutations on the main functional properties of the EAATs. These include transporter/exchanger functionality, the cation and substrate affinities, the Na^+^/Li^+^ selectivity, and the ability to interact with K^+^ ions [4]–[26]. We will refer to this table to motivate the choices made in modeling of EAAT3 and justify the simulation results.

**Table 1:** Effect of different mutations on the properties of EAAT1, EAAT2 (GLT-1) and EAAT3 (EAAC1) transporters.

Transporter		Trans.	Exch.	Na^+^ aff.(s)	Na^+^ aff.	K^+^ int.	Li^+^ int.	Glu aff.*	Asp aff.*
EAAT1	Wild-Type [4], [5]	Yes	Yes	20 mM		Yes	Yes	24 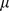 M	16 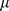 M
	T131A [6]		Yes	N/S				1Red	
	S363R/R477M [4]	No	Yes	0Inc		No		2Inc	2Inc
EAAT2	Wild-Type [7]–[10]	Yes	Yes	24 mM		Yes	No	5.3 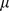 M	2.8 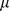 M†
	Y403F [9]	No	Yes	1Inc		No	Yes	WT	WT†
	E404D [7]	No	Yes	WT		No			
	S440G [10]	Yes	Yes			Yes	Yes	WT	WT†
EAAT3	Wild-Type [11]–[13]	Yes	Yes	8 mM	120 mM	Yes	Yes	14 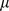 M	14 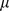 M
	T101A [14]	Yes	Yes	0Red	N/S	Yes		2Red	
	H296Q [15]	Yes	Yes	WT		Yes		WT	
	N366Q [16]	Yes	Yes	N/S		Yes	No	3Red	2Red
	D368E [16]	Yes	Yes	WT		Yes	No	None	1Red
	D368N [13]	No	Yes	0Red	N/S	No		2Red	
	N366D/D368N [16]	No	Yes			No	No	1Red	0Red
	T370S [12], [17], [18]	Yes	Yes	N/S	0Red	Yes	No	2Red	WT
	E374Q [11], [19]	Yes	Yes	WT	WT	Yes		WT	
	D440N [11]	No	No	1Red	WT			2Red	1Red
	D440E [20]	Yes	Yes	1Inc		Yes	Yes	1Inc	1Inc
	D444C [21]	No	Yes‡					WT	WT
	D444E [21]	No	Yes	0Inc				WT	WT
	R445E [21]	Yes	Yes						
	R445S [22]	Yes	Yes	WT			Yes	1Inc	1Inc†
	R447C [23]	No	No	Becomes a Na^+^ dependent serine/cysteine exchanger					
	N451S [17]	Yes	Yes	N/S		Yes	No	2Red	1Red
	D455N [11], [13], [24], [25]	No	Yes	WT	WT	Yes	Yes	0Inc	0Inc
	D455S [24]	No	Yes	0Red		No	Yes	WT	0Inc
	D455E [24]	No	No						
	D455A [24]–[26]	No	No		WT				

Transporter/Exchanger functionality is given in the third and fourth columns. Columns 5 and 6 gives the Na^+^ affinity in the presence and absence of glutamate, respectively. Columns 7 and 8 specify K^+^ and Li^+^ interactions with the transporter. Glu and Asp affinities are given in the last two columns. For the affinities, N/S means no saturation of the binding site in the range considered, and nRed/nInc means reduction/increase in the ligand affinity by 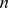 orders of magnitude (0, same order). Human sequences are used for EAAT3 which have one extra residue than the rat sequences employed in some experiments.

*All glutamate affinities measured at [Na^+^] between 100–150 mM.

† Result for D-aspartate.

‡ Enables succinate exchange, unlike wild-type.

A recent breakthrough in our understanding of the EAATs came with the crystal structure of the archaeal homolog Glt_Ph_ [27], and the identification of the Asp substrate and two of the Na^+^ binding sites (Na1 and Na2) [28]. These initial structures captured Glt_Ph_ in the outward-facing state. The inward-facing state was similarly characterized in a subsequent crystal structure [29]. Glt_Ph_ shares about 37% sequence identity with the EAATs, but the homology is much higher for residues near the binding pocket, reaching around 60% in this region. Furthermore, almost all of the residues shown to be involved in the binding of the ions and aspartate in Glt_Ph_ are conserved in the EAATs [23], [27]–[31]. Experiments have shown that, while the transport mechanism in Glt_Ph_ is independent of H^+^ and K^+^ ions [32], it is still coupled to the co-transport of three Na^+^ ions as in EAATs [33]. The third Na^+^ site (Na3) could not be identified in the crystal structures of Glt_Ph_, but a binding site determined from molecular dynamics (MD) simulations was confirmed from mutagenesis experiments and shown to be conserved in Glt_Ph_ and the EAAT family [6]. Because of its structural and functional similarity to EAATs, Glt_Ph_ provides a good model for studying the transport mechanism in EAATs [34], [35]. This has prompted several MD simulations of Glt_Ph_, investigating the gating mechanism in the outward [36]–[38] and inward-facing states [38]–[40], location of the Na3 site [6], [18], [41], binding free energies of the ligands [18], [40], [42], and substrate translocation [43]–[45].

In order to facilitate homology modeling of EAATs from Glt_Ph_, we list in Table 2 all the functionally important residues close to the binding site, which are identified from the crystal structures and the mutation experiments in Table 1. Considering the conserved residues first, there seems to be good agreement between the crystal structure data and mutagenesis experiments, e.g., identification of D444 and R447 in EAAT3 as being involved in the coordination of glutamate 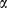-amino and side-chain carboxyl groups, respectively [21], [23]. There is, however a discrepancy related to D455 in EAAT3, which corresponds to D405 in Glt_Ph_. In Glt_Ph_, D405 coordinates the Na1 ion with both side-chain carboxyl oxygens. But the D455N mutation in EAAT3 has shown little effect on the Na^+^ affinity both in the presence and absence of the substrate (Table 1). To resolve this issue, it has been proposed that D455 might be protonated during the whole transport cycle, which would explain why neutralizing this residue does not affect the Na^+^ affinity [25]. Another important difference between EAATs and Glt_Ph_ is the K^+^ binding site in the inward-facing state of EAATs, which is not present in Glt_Ph_. Different sites have been suggested for K^+^ binding, overlapping with either the proposed protonation site [7], or the substrate 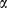-amino group [46], or the Na1 site [24], [25].

**Table 2:** Glt_Ph_ residues involved in the coordination of the ligands and their equivalents in EAAT1, EAAT2 and EAAT3.

Glt_Ph_	Y89	T92	S93	**Q242**	**R276**	S277	S278	G306	T308
EAAT1	Y127	T130	T131	**H328**	**S363**	S364	S365	G394	T396
EAAT2	Y124	T127	T128	**H326**	**A361**	S362	S363	G392	T394
EAAT3	Y98	T101	T102	**H296**	**S331**	S332	S333	G362	T364
Glt_Ph_	N310	D312	T314	Y317	**Q318**	S349	I350	**T352**	**G354**
EAAT1	N398	D400	T402	Y405	**E406**	S437	I438	**A440**	**G442**
EAAT2	N396	D398	T400	Y403	**E404**	S435	I436	**A438**	**S440**
EAAT3	N366	D368	T370	Y373	**E374**	S405	I406	**A408**	**G410**
Glt_Ph_	V355	G359	D390	D394	**M395**	R397	T398	N401	D405
EAAT1	I443	G447	D472	D476	**R477**	R479	T480	N483	D487
EAAT2	I441	G445	D470	D474	**R475**	R477	T478	N481	D485
EAAT3	V411	G415	D440	D444	**R445**	R447	T448	N451	D455

Residues from the mutagenesis experiments in Table 1 are also included in the table. Those residues that are not conserved between Glt_Ph_ and EAATs are indicated with boldface. Human sequences are used for EAAT3.

There are only six non-conserved mutations between Glt_Ph_ and EAATs in Table 2. Of these, R276 and T352 contribute backbone carbonyls to the coordination of the Glu substrate and the Na2 ion, respectively, while the others are not directly involved in ligand coordination. Mutation of R276 and M395 in Glt_Ph_ to S331 and R445 in EAAT3 switches the position of the arginine residue from the HP1 segment in Glt_Ph_ to the TM8 segment in EAATs, which will be discussed later in the paper. The Q242 residue in Glt_Ph_ is a histidine in EAATs, which has been proposed as the protonation site in EAATs. But this was contradicted by the H296Q mutation in EAAT3, which had no effect on the transport mechanism (Table 1). The Q318 residue in Glt_Ph_ is a glutamate in EAATs, suggesting that this might be the protonation site. There is strong experimental evidence for this hypothesis, e.g., the E374Q mutation does not affect the Glu affinity of EAAT3 (Table 1), but it abolishes the pH dependence of Glu translocation seen in the wild-type transporter [19], [47]. Another piece of evidence comes from the neutral amino acid transporters, where the corresponding residue is also a glutamine like in Glt_Ph_, and the pH sensitivity can be engineered into these transporters by mutating this glutamine to glutamate [19]. The G354 residue in Glt_Ph_ is conserved in EAAT1 and EAAT3, but it is a serine in EAAT2. Among EAATs, EAAT2 is the only one where Li^+^ cannot replace Na^+^ in the transport cycle. Either the S440G or Y403F mutation enables Li^+^ to support transport in EAAT2, indicating that the S440 and Y403 residues are involved in the Na^+^/Li^+^ selectivity of EAAT2 [10].

Here we attempt to address the issues raised above by performing MD simulations on two homology models for EAAT3 based on the Glt_Ph_ transporter in the inward- and outward-facing states. We analyze several properties of EAAT3 such as the coordination of the ligands and the stability of the substrate with a protonated/deprotonated E374 side chain, the effect of the D455N mutation (or protonation of D455) on Na^+^ affinity, and the extracellular (EC) and intracellular (IC) gating mechanisms. We also discuss binding of a K^+^ ion in the inward-facing state and propose a binding site based on free energy calculations.

## Results and Discussion

### Model Description

We have used the crystal structures of Glt_Ph_ in the outward- (PDB ID 2NWX) and inward-facing states (PDB ID 3KBC) to construct two homology models for the EAAT3 transporter. Both conformations have the substrate binding site occluded from the solvent, and have an Asp and two Na^+^ ions bound (Na1 and Na2). To build the models, we have used the program MODELLER [48] and the alignment from Ref. [27]. The templates had Asp, Na1, Na2 and Na3 bound, with this last Na^+^ added at the site as described in Ref. [6]. We have created the models including the ligands in the alignment in order to obtain EAAT3 structures in the fully-bound states. We have created 20 models for each conformation and assessed the quality of the structures by using the Swiss Model Server [49], [50]. After choosing the best models, we have constructed a trimer for each conformation by superposing the EAAT3 monomer to chains A, B and C of Glt_Ph_. These two models have been employed in the MD simulations.

EAAT3 has a sequence of around 50 residues close to the trimerization domain, called 4B–4C loop, which is not present in Glt_Ph_. In order to ensure robustness of EAAT3 trimers, we had to exclude this region from the models. Experiments have shown that cysteine substitution in the residues from this loop has little effect on the transporter properties [51]. However, studies of the LacY transporter indicate that this does not necessarily mean that this segment is not important for transport, as it can be involved in conformational changes happening during the transport cycle [52]. To make sure that the truncation of this segment has little effect on our results, we have also built outward and inward models with the inclusion of the 4B–4C loop. This is achieved by placing the 4B–4C loop in the position suggested from FRET studies [51], and adding restraints in MODELLER (see Figure S1 in File S1). It can be seen from Figure S1 that this loop is not close to the conformational changes we investigate in this study or to the bound ligands, which corroborates our approach. While this full model of EAAT3 is sufficient to give an idea about the distance between the 4B–4C loop and the binding pocket, there are large uncertainties in the secondary structure of the 4B–4C loop, which may cause simulation artifacts. Therefore, we prefer to use the truncated model without the 4B–4C loop in the MD simulations. Regarding the other segments of EAAT3, a previous FRET study has shown that their relative positions have a strong correlation with the Glt_Ph_ crystal structure, which indicates that the structures are well conserved between the two transporters [53].

### Evaluation of the Models

We have assessed the quality of our EAAT3 homology models using three different methods. QMEAN (Qualitative Model Energy ANalysis) is a composite scoring function ranging from 0 to 1, which describes the major geometrical aspects of protein structures [50]. DFIRE (Distance-scaled, Finite Ideal-gas REference) is a potential based on a database of non-homologous proteins [49]. DOPE (Discrete Optimized Protein Energy) is an atomic distance-dependent statistical potential calculated from a sample of native structures [48]. The first two are available on the Swiss Model Server, and the last one is built into the program MODELLER. In Table 3 we show the QMEAN scores and DFIRE energies for the original Glt_Ph_ crystal structures, as well as our EAAT3 models in the outward and inward-facing states. It is seen that the QMEAN and DFIRE values are comparable between the crystal structures and the EAAT3 models, which shows that the EAAT3 models are reliable, and in principle provide a good option to investigate EAATs. The DOPE energy profile for the outward-facing EAAT3 model is compared with that of the 2NWX crystal structure in Figure S2A in File S1, and the same is done for the inward-facing EAAT3 model and the 3KBC crystal structure in Figure S2B in File S1. The profiles are very similar, especially in the regions that form the glutamate binding site, which range from residues 260 to 410. We have also compared the Ramachandran plots for the templates and the models, which agree very well with few residues in the forbidden regions in all cases (Figure S3 in File S1). These results confirm that, even though the homology between EAAT3 and Glt_Ph_ is not very high, we can still build good models and hopefully gain insights into the mammalian glutamate transporters using the structural information from the archaeal homolog.

**Table 3:** QMEAN scores and DFIRE energy values for the Glt_Ph_ crystal structures, and the EAAT3 models constructed in this work.

Structure	QMEAN score	DFIRE Energy
Glt_Ph_ (2NWX)	0.524	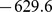
EAAT3 (outward)	0.489	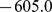
Glt_Ph_ (3KBC)	0.557	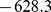
EAAT3 (inward)	0.540	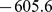

The QMEAN score goes from 0 to 1, 1 being the best possible model. Lower DFIRE energy values indicate better quality structures.

### Fully Bound EAAT3

We first discuss the stability of the ligands in the outward and inward fully-bound models of EAAT3 with the EC-gate and IC-gates closed. To check the hypothesis that E374 is the proton carrier in EAAT3, we have prepared two systems for the outward-facing state of EAAT3: one with the side chain of E374 protonated and the other with a deprotonated E374 side chain in all three monomers. All of these systems have been equilibrated for 20 ns initially. In order to show that our models are stable for longer periods, we have performed an additional 40 ns of MD simulations for the outward and inward fully bound states with protonated E374. The RMSDs of the protein backbone for these models, obtained from the 60 ns MD data, are shown in Figure S4 in File S1. Both RMSDs show no major changes after the first 10 ns. More importantly, the coordination of the ligands remain intact, exhibiting no perceptible changes between 10–20 ns and 20–60 ns of data collection. These results indicate that our systems are stable in the vicinity of the outward and inward states, and 20 ns appears to be sufficient for their equilibration.

**Figure 2:**
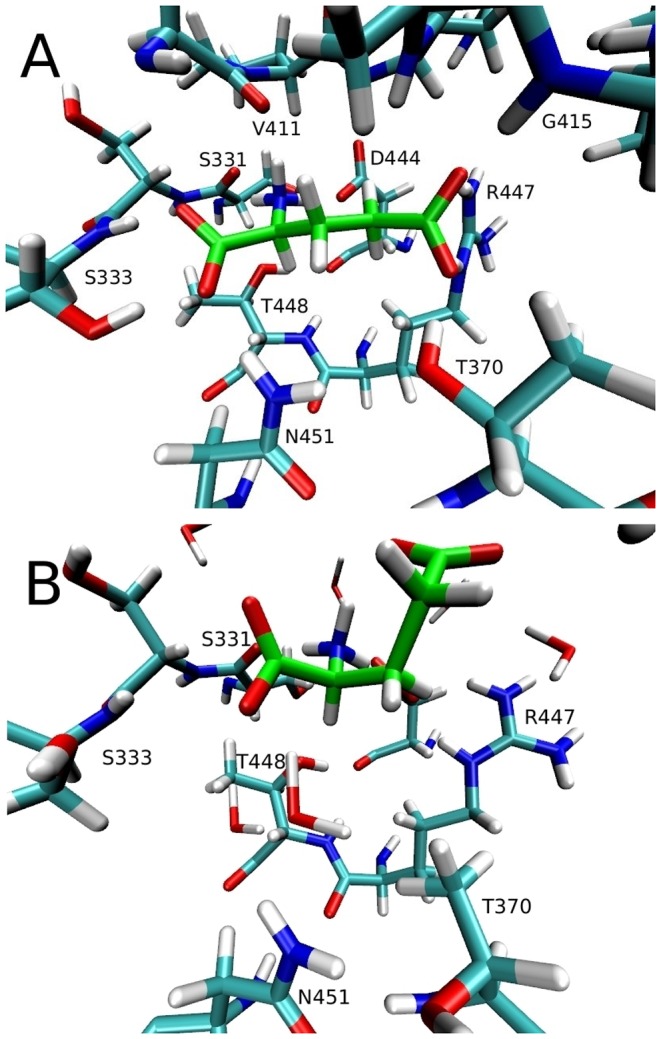
Binding of glutamate to EAAT3 with a protonated vs deprotonated E374 side chain. (A) Glutamate substrate (in green) bound to EAAT3 in the closed outward state with a protonated E374 side chain. (B) The same but with a deprotonated E374 side chain. Glutamate is stable in A for 20 ns, but becomes unstable in B, losing most of the contacts after 10 ns of simulations.

Our simulations with the protonated E374 residue show that Glu is stable, remaining bound to the transporter with the EC-gate closed and the coordination shown in Table 4 throughout the 60 ns of MD simulations (Figure 2A). We have applied the same procedure in the inward-facing state of EAAT3, and obtained very similar results for the coordination of Glu when the E374 side-chain is protonated (Table 4). In contrast, if we have a deprotonated E374 side-chain, the substrate shows instability and loses most of its contacts with the transporter (Figure 2B). In the closed state, the substrate and the E374 side-chain carboxyl groups are separated by around 7 Å, and there are no water molecules between them so the dielectric screening is expected to be small in this region. Thus the electrostatic repulsion between these two groups is likely to destabilize Glu in the binding site, making the protonation of E374 necessary for Glu binding. This is consistent with the experimental observations indicating that protonation of E374 precedes Glu binding [19], [47]. We have further tested this possibility by performing 20 ns of simulations of the Glt_Ph_ Q318E mutant in two systems previously equilibrated in a recent study [42]. One of them had Asp, Na1, Na2 and Na3 bound in the outward-facing closed state, and the other one had Asp, Na1 and Na3 bound in the outward-facing open state. In the first case we don't see any changes, except for the partial hydration of the E318 residue that takes place in one of the chains after a few nanoseconds. In the second, however, we see that the Asp substrate becomes unstable and loses most of its contacts with the protein, and Asp is released back to the solvent in two of the three chains of Glt_Ph_. A movie is included in File S2, showing the release of the Asp substrate in one of the chains that occurs between 5 and 15 ns.

**Table 4:** List of the EAAT3 residues coordinating the Glu substrate atoms.

	Glu-	Outward	Inward
Helix-Residue	atom	EAAT3	EAAT3
HP1 - S331 (O)	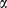 -N	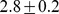	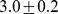
HP1 - S333 (N)	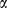 -O_1_	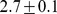	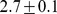
HP1 - S333 (OH)	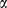 -O_2_	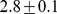	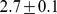
TM7 - T370 (OH)	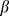 -O_2_	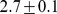	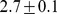
HP2 - V411 (O)	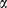 -N	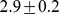	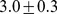
HP2 - G415 (N)	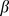 -O_1_	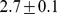	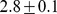
TM8 - D444 (O_1_)	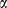 -N	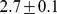	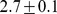
TM8 - R447 (N_1_)	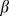 -O_1_	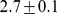	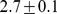
TM8 - R447 (N_2_)	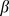 -O_2_	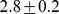	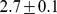
TM8 - T448 (OH)	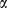 -N	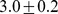	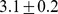
TM8 - N451 (N*_δ_*)	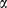 -O_2_	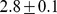	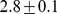

The average N–O and O–O distances (in Å) are obtained from 5 ns of MD simulations in the closed conformation of the outward and inward-facing states of the transporter. Bare O and N atoms in parenthesis refer to the backbone atoms and the others with subscripts refer to the side chain atoms.

Regarding the ions in our EAAT3 models, Na1 and Na3 are very stable and have very similar coordinations compared to those in Glt_Ph_ [40], [42] (Table 5). This is expected because all of the residues in the coordination shell of both ligands are conserved between EAAT3 and Glt_Ph_, except for T102 which is a serine (S93) in Glt_Ph_. The Na2 ion, on the the other hand, is unstable and spontaneously unbinds in two of the chains in the outward-facing state and one of the chains in the inward-facing state. The ones that remain bound are coordinated by the residues shown in Table 5. Because the Na2 site in the crystal structure has contacts with the backbone carbonyl oxygens, it is difficult to assess it through mutagenesis experiments. Previous MD studies of Glt_Ph_ have shown that this site is unstable [54], which is consistent with our observations. It is possible that the Na2 site is not conserved in EAATs. Mutagenesis experiments have shown that the T370S [17], [18] and the N451S [17] mutations seem to affect the Na^+^ affinity and selectivity in EAAT3 (Table 1), and a site having the coordination of these residues and the substrate has been suggested [18]. Since Na2 is likely to be the only Na^+^ ion to bind to EAAT3 after the substrate, this site would make a good candidate for the Na2 site if the original site from the Glt_Ph_ crystal structure is shown not to be conserved in EAATs. More experiments and simulations are needed to clarify this issue.

**Table 5:** List of the EAAT3 residues coordinating the three Na^+^ ions.

		Outward	Inward
	Helix-Residue	EAAT3	EAAT3
Na1	TM7 - G362 (O)	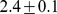	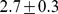
	TM7 - N366 (O)	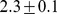	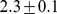
	TM8 - N451 (O)	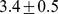	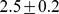
	TM8 - D455 (O_1_)	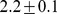	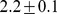
	TM8 - D455 (O_2_)	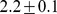	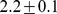
	H_2_O	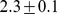	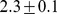
Na3	TM3 - Y98 (O)	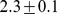	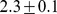
	TM3 - T101 (OH)	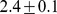	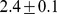
	TM3 - T102 (OH)	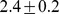	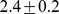
	TM7 - N366 (O*_δ_*)	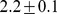	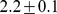
	TM7 - D368 (O_1_)	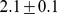	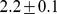
	TM7 - D368 (O_2_)	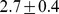	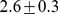
Na2	TM7 - T364 (OH)	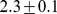	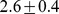
	HP2 - I406 (O)	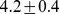	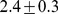
	HP2 - A408 (O)	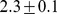	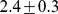
	H_2_O	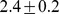	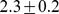

The average Na^+^–O distances (in Å) are obtained from 5 ns MD simulations of the closed state in the outward and inward-facing conformations of EAAT3. Nature of the coordinating oxygens are indicated in parenthesis.

### Opening of the EC- and IC-Gates

**Figure 3:**
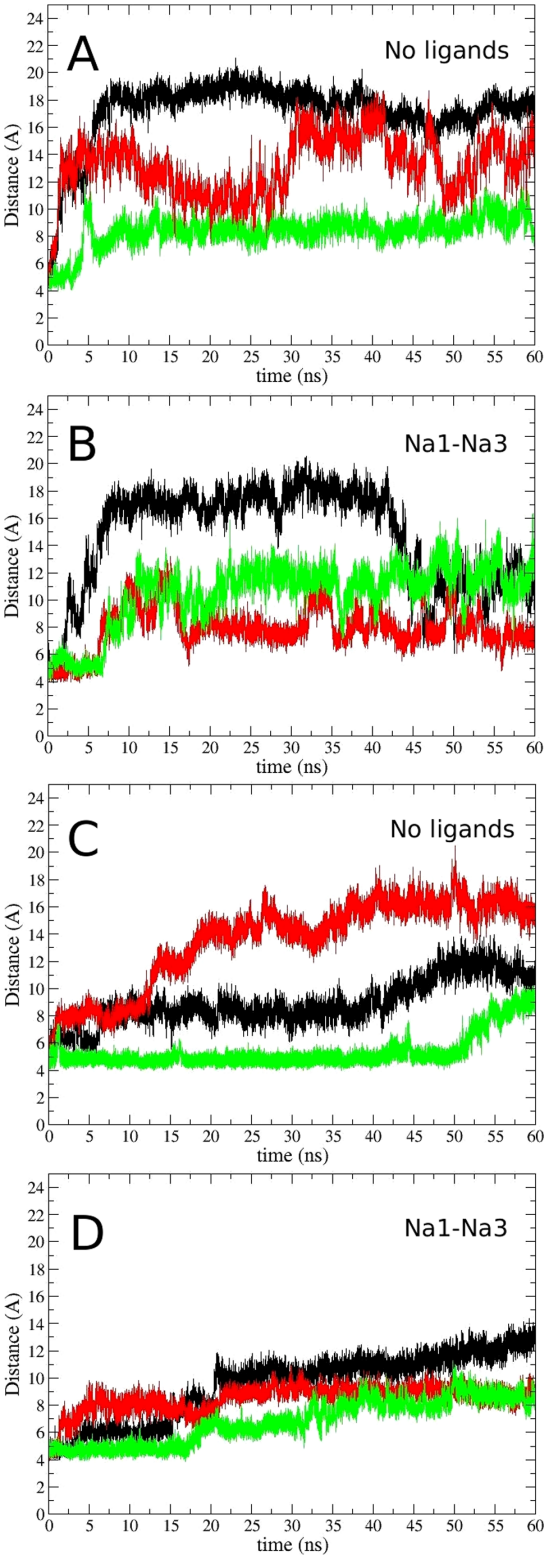
Gating motions in the outward- and inward-facing conformations of EAAT3. Time evolution of the S332(C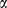)–V411(C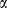) distance in the outward-facing conformation with (A) no ligands bound to the transporter, and (B) only the Na1 and Na3 ions bound to the transporter. The same for the inward-facing conformation with (C) no ligands bound to the transporter, and (D) only the Na1 and Na3 ions bound to the transporter. The color code for the subunits are A (black), B (red), and C (green).

In order to observe the gating mechanism in EAAT3, we have performed two 60 ns simulations for each model: one of them had no ligands bound, and the other had only the Na1 and Na3 ions bound (Figure 3). The opening of the EC- and IC-gates is monitored using the S332(C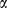)–V411(C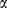) distance, which has been used in previous studies of the Glt_Ph_ transporter [37], [40]. These two residues are located at the tips of the HP1 and HP2 hairpins, respectively, and hence provide a good measure for the opening of the EC-gate. In the simulations of the fully-bound state this distance remains around 5 Å, meaning that the gates are closed in this configuration. Once the Glu substrate is removed, both the EC- and the IC-gates show opening in all of the chains regardless of whether the Na1 and Na3 ions are present or not (Figure 3). Presence of the Na^+^ ions appears to reduce the fluctuations in the gate motions but does not have much effect on the opening of the gates. In both Figures 3B and 3D, the Na1 and Na3 ions are not affected by the gate opening despite the flooding of the binding site by water molecules, and remain bound with the coordination shown in Table 5. This shows that these sites are very stable in EAAT3 even after 60 ns of simulations in the absence of the substrate, so they are likely to be the two Na^+^ ions that are proposed to bind to EAATs before glutamate binds [14].

**Figure 4:**
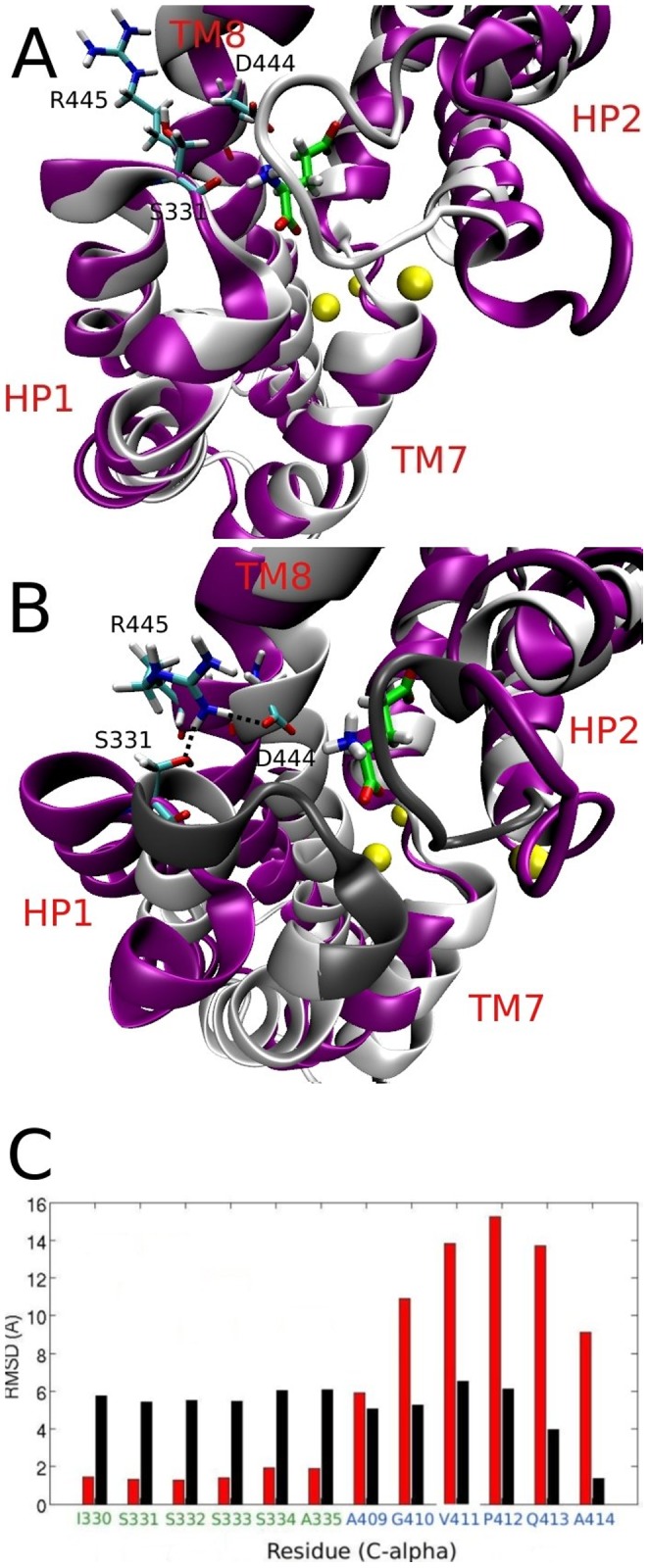
Comparison of gate opening in the outward- and inward-facing conformations of EAAT3. (A) Overlap between the closed (grey) and the open (purple) states in the outward EAAT3. Glutamate and three Na^+^ ions bound to the transporter, as well as the S331, D444 and R445 residues are indicated. (B) The same for the inward-facing state, showing a large movement of HP1, HP2 and TM8. The contacts made by the insertion of the R445 side-chain between HP1 and TM8 are also shown. The dark shade of grey in the closed state indicates the position of the residues from part C below. (C) Comparison of the IC-gate opening (black) with the EC-gate opening (red). The RMSD of the C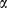 atoms at the tips of HP1 and HP2 is obtained by aligning the open and closed states and calculating the RMSD from 2 ns of MD simulations.

To assess in more detail the conformational changes brought by the gate opening in the outward and inward-facing states, we choose the chains that show the largest opening in the absence of ligands in both cases, which are chain A and chain B, respectively, in Figures 3A and 3C. In Figures 4A and 4B, we show the overlap of these two structures with their respective closed states, and in Figure 4C we show the average RMSD displacement of the C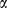 atoms of six residues located at the tips of each of the two hairpins. These are calculated by aligning each frame and calculating the RMSD of each atom between the closed and open states during 2 ns of MD simulations. The opening observed in the outward facing EAAT3 looks very similar to Glt_Ph_, with a large movement of HP2 and a small displacement of the HP1, TM7 and TM8 segments. The inward state, on the other hand, shows movements of HP1, HP2 and TM8, with a much larger opening of HP1 compared to our previous study of Glt_Ph_ [40]. From Figure 4B, we see that this is caused by the insertion of the R445 side-chain in between the residues S331 and D444, making contacts with them. This shifts the HP1 and TM8 segments apart, causing a larger exposure of the binding site. This event is observed at around 40 ns in the simulation of Figure 3C, but the contacts are kept and the transporter maintains the large opening in the remainder of that simulation. Interestingly, Glt_Ph_ has a methionine in the position equivalent to R445, and an arginine replaces the serine in position 331.

It is instructive to compare our results to those obtained from metadynamics simulations on Glt_Ph_ by Grazioso et al. [38]. In both cases, opening of the gate in the outward-facing state shows a large movement of HP2, and a small displacement of the neighbouring segments. Similarly, during the opening of the gate in the inward-facing state, both studies show a movement of not only HP1 and HP2, but also of the nearby segments TM7 and TM8. A salt bridge between R276 and D394 is observed in the inward open state of Glt_Ph_, which is similar to the R445–D444 salt bridge we see in our simulations. We also compare our results for EAAT3 to the microsecond MD simulations of Zomot and Bahar [39] in the inward-facing state of Glt_Ph_. In Glt_Ph_, a salt bridge between D394 and R276 prevents HP1 from opening further, but it also attracts the Glu substrate away from the binding site, facilitating its release. In EAAT3 there is still a salt bridge in this position, but it has the opposite effect on the gating; it allows the transporter to open further and stabilizes an open conformation through the contact between R445 and D444. Therefore, the switching of this arginine from position 331 to 445 in EAAT3—which is conserved in all EAATs—has a two-fold effect: first, it creates a more stable and open conformation of the transporter in the inward state, which facilitates the release of the larger Glu substrate; and second, it conserves the salt bridge (R445–D444) in the vicinity of the binding site that has been shown to help the release of the substrate. Even though this is an intermediate state followed by further conformational changes in the transporter, these are significant features of inward gating that are worth emphasizing.

### The D455N Mutation

As mentioned in the Introduction, there is an apparent disagreement between the crystal structure and the mutagenesis experiments concerning the Na1 binding site, raising the possibility that the D455 residue is protonated throughout the transport cycle. To address this issue, we have prepared four simulation systems in the outward-facing state of EAAT3: two systems with the D455N mutation and with either Na1 and Na3 bound or with only Na1 bound, and another two with a protonated D455 residue and the same Na1/Na3 occupancy as above. For the Na1 and Na3 bound systems, we use the last frame of the simulation in Figure 3B. The Na1 bound system is prepared by removing Na3 from this system and equilibrating it for a further 10 ns. We perform a 2 ns alchemical transformation in the D455 side-chain to an asparagine or to a protonated aspartate, and at the same time gradually release restraints applied to the Na1 ion. After the mutation/protonation step, we equilibrate the system for a further 8 ns, monitoring the distance between the side-chain carboxyl/carboxamide carbon atom and the Na1 ion.

**Figure 5:**
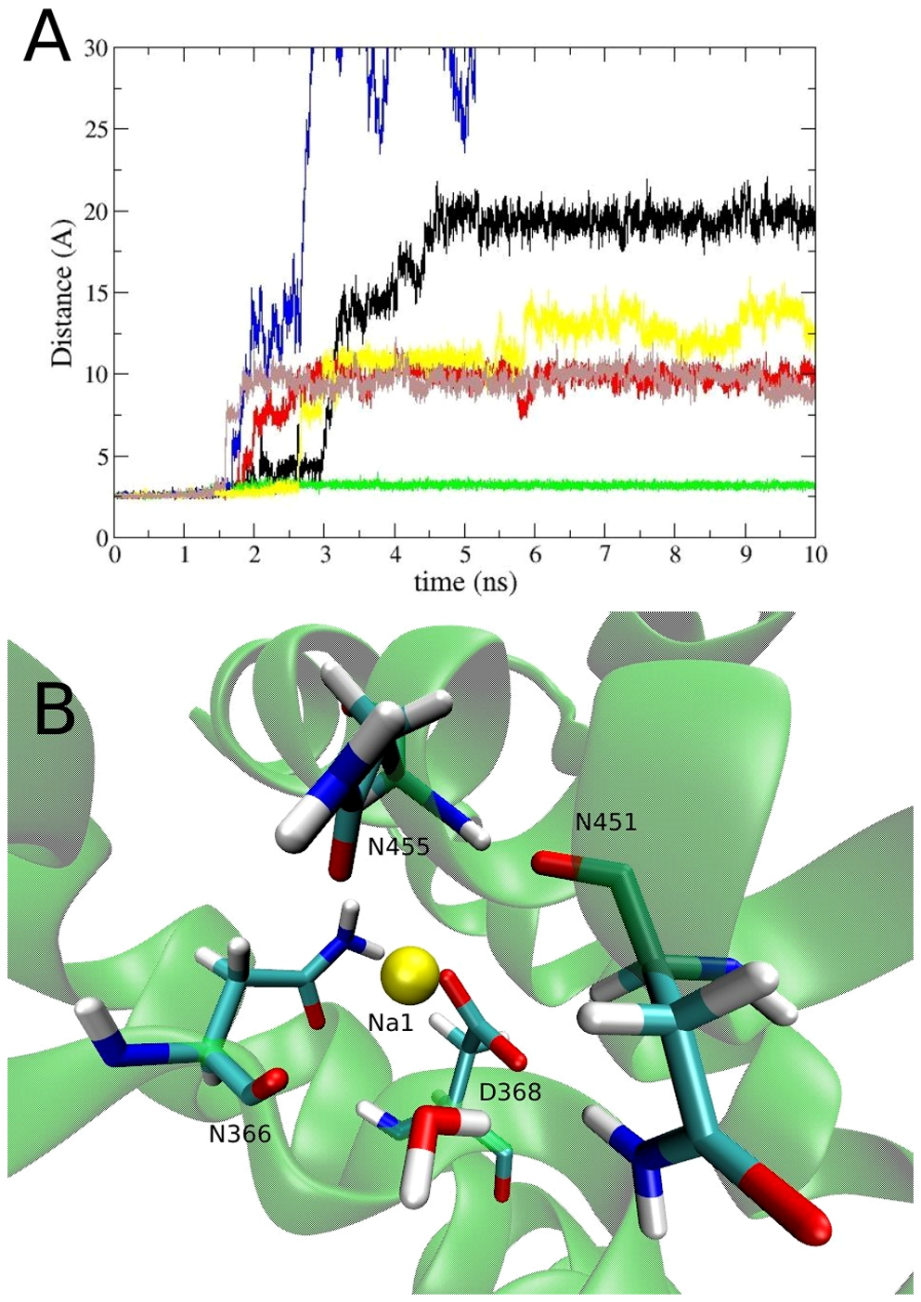
Effect of the D455N mutation on sodium binding. (A) The distance between the Na1 ion and the side-chain carboxyl/carboxamide carbon atom of the residue D455 during its mutation from aspartate to an asparagine (black, red and green), and to a protonated aspartate (blue, yellow and brown) in the presence of the Na3 ion. In the first case, Na1 leaves the binding site in 2 out of 3 chains, and in the second case, Na1 leaves the binding site in all chains. (B) The new coordination of Na1 when the D455N mutation is performed in the absence of the Na3 ion. This site has the coordination of the carbonyl oxygens of N366 and N451, as well as the side chain oxygens of D368 and N455.

The results for the D455N mutation and the D455 protonation in the presence of Na3 are displayed in Figure 5A. It is seen that Na1 is released to the solvent in all of the chains after the protonation of D455, and in two out of three chains after the D455N mutation. Therefore, it is unlikely that EAAT3 can support the binding of both Na1 and Na3 if the D455 side-chain is protonated. When we perform the same simulations in the absence of the Na3 ion, Na1 is found to be stable in all the chains for the whole 10 ns (not shown). In this case, we observe that the Na1 ion finds a new coordination shell involving the carboxyl oxygens of D368, one carboxamide/carboxyl oxygen of N455/D455 and the carbonyl oxygens of residues N366 and N451 (Figure 5B). This site involves some of the residues that coordinate the original Na1 ion (Table 5), and is located about 3 Å from the Na1 site. In the absence of the Glu substrate, the Na^+^ affinity is measured via the [Na^+^]-dependent anion leak current, which is unaffected by the D455N mutation in EAAT3. This may be explained by assuming that this current is coupled to the binding of two Na^+^ ions in the wild-type transporter, but only to one Na^+^ ion after the D455N mutation. It is important to mention that, even though the D455N mutation does not affect the Na^+^ or Glu affinity in EAAT3, it does lock this transporter in the exchange mode (Table 1), meaning that one of the steps in the transport cycle is impaired by this mutation.

### The Potassium Binding Site

**Figure 6:**
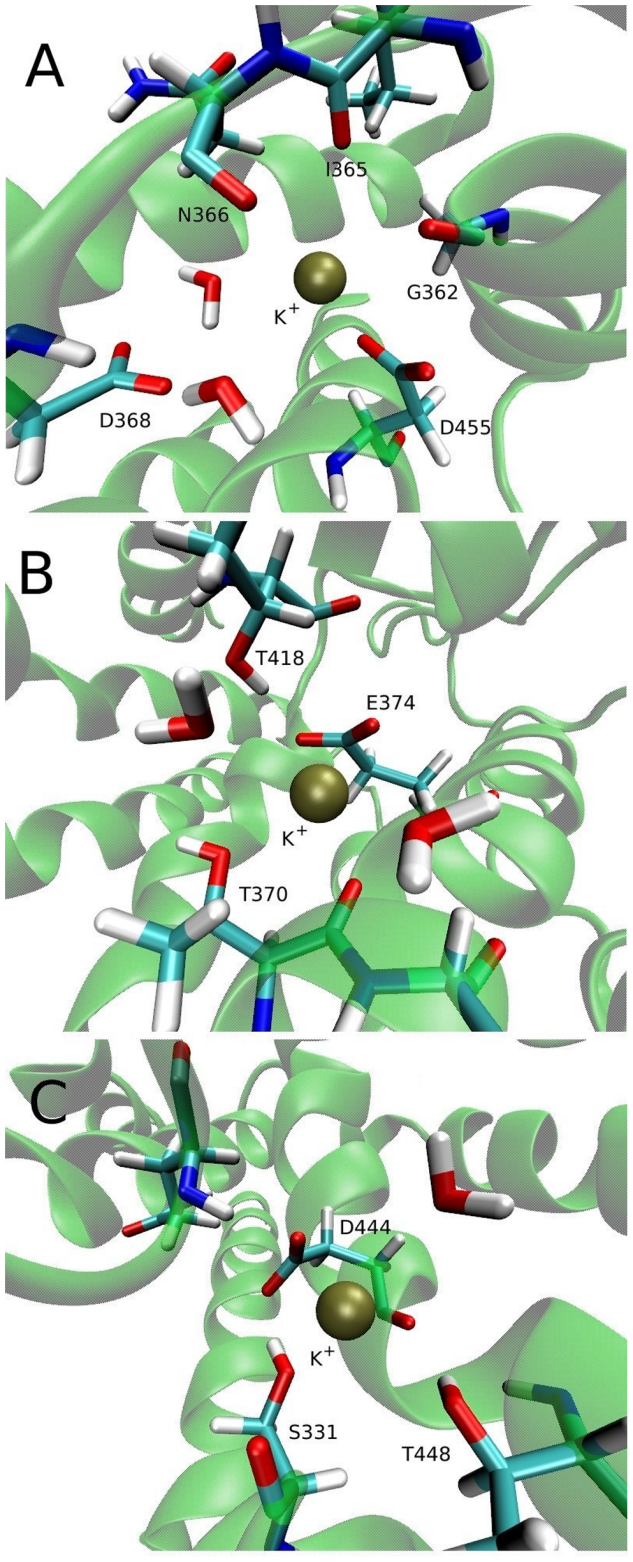
Three potential sites for binding of a K^+^ ion. (A) Site 1 is obtained by replacing Na1 by a K^+^ ion and equilibrating the system for 10 ns in the absence of other ligands (B) Site 2 is obtained by restraining K^+^ close to the E374 carboxyl group and equilibrating the system for 10 ns with no other ligands bound. (C) Site 3 is obtained by placing a K^+^ ion near glutamate 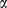-N atom and equilibrating the system for 10 ns in the absence of other ligands.

In contrast to Glt_Ph_, EAATs counter-transport one K^+^ ion back to the extracellular medium at each cycle. Binding of K^+^ is essential for the reorientation of the transporter back to the outward-facing state after the release of all the ligands into the intracellular medium. It has been suggested that mutants that are locked in the exchange mode cannot interact with K^+^, and therefore are not able to carry out the second half of the transport cycle. This is the case for a few mutations close to the Na1 and Na3 binding sites, such as the D368N, D455A, D455S mutations and the N366D/D368N double mutation in EAAT3 (Table 1). The D455N mutation is an exception where the transporter is still locked in the exchange mode, but it could interact with K^+^ albeit with a reduced affinity compared to wild type [25]. Therefore, this region is a good candidate for the K^+^ binding site in EAAT3, which we call site 1. To obtain this site, we remove the Na3 ion from the last state of the simulation in Figure 3D—where EAAT3 is in the Na1-Na3 bound inward-facing state—replace the Na^+^ ion at Na1 to K^+^ in all of the three chains and equilibrate the system for a further 50 ns. The final coordination of K^+^ is shown in Figure 6A, which is similar in all of the three subunits. Site 1 makes contacts with both side-chain oxygens of residue D455, the backbone atoms of residues G362, I365, and N366, and two water molecules (see Table 6 for the contact distances). The two water molecules are very stable as they are kept in place by the D368 side-chain, which is also close to the K^+^ ion. This region has a strong negative potential due to the presence of two negatively-charged side chains, and therefore is likely to harbour a cation binding site.

**Table 6:** EAAT3 residues coordinating the K^+^ ion at sites 1, 2, and 3.

	Helix-Residue	Distance
site 1	TM7 - G362 (O)	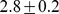
	TM7 - I365 (O)	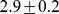
	TM7 - N366 (O)	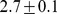
	TM8 - D455 (O_1_)	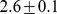
	TM8 - D455 (O_2_)	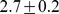
	H_2_O (1)	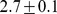
	H_2_O (2)	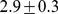
site 2	TM7 - T370 (OH)	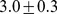
	TM7 - T370 (O)	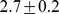
	TM7 - E374 (O_1_)	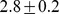
	TM7 - E374 (O_2_)	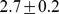
	H_2_O (1)	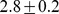
	H_2_O (2)	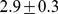
site 3	HP1 - S331 (O)	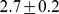
	HP1 - S331 (OH)	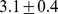
	TM8 - D444 (O)	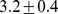
	TM8 - D444 (O_1_)	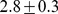
	TM8 - D444 (O_2_)	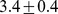
	TM8 - T448 (OH)	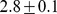
	H_2_O	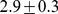

Notation is the same as in Table 5.

In EAAT2, the Y403F and E404D mutations also lock this transporter in the exchange mode (Table 1). The same happens for the E374Q mutation in EAAT3, even though in this case the transporter is still able to interact with K^+^ in the homoexchange mode [19]. This raises the possibility that the K^+^ binding site might overlap with the proton binding site, which we call site 2. To obtain this site, we restrain a K^+^ ion close to the deprotonated E374 side-chain and equilibrate the system in the absence of other ligands for 50 ns, gradually releasing the restraints applied to the K^+^ ion. The K^+^ ion remained in a similar position in two of the three chains and was unstable. The one that has the tightest coordination is chosen for site 2 (Figure 6B), and will be used in free energy calculations. The contacts in site 2 are the two side-chain oxygens of E374, and both the backbone oxygen and the side-chain hydroxyl group of T370 (Table 6). While the T418 residue does not directly interact with K^+^, it remains close to this ion and might have an indirect effect on the stability of K^+^ in site 2.

The last site we consider (to be called site 3) overlaps with the positively-charged substrate 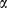-amino group and was proposed from electrostatic mapping calculations of a homology model of EAAT3 [46]. This site has also been suggested in a recent study of the novel mosquito dicarboxylate transporter CuqDCT, which has an asparagine at the position equivalent to the D444 residue in EAAT3 [55]. Mutations of this residue or its equivalent affect K^+^ counter-transport in both EAAT3 and CuqDCT, so this site is also a good candidate for binding of a K^+^ ion. Here we obtain this site by removing the substrate and placing a K^+^ ion in the same position as its 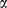-amino N atom. The system is then equilibrated for 50 ns in the absence of other ligands. The same coordination is obtained for site 3 in all chains of EAAT3 (Figure 6C), which is also very similar to the one suggested in Ref. [46]. Site 3 has contacts with an oxygen of the D444 side-chain, the hydroxyl groups of S331 and T448, and the backbone atoms of D444 and S331 (Table 6). Even though site 3 has many coordinating residues, it is also close to the positively-charged R445 and R447 residues, which will reduce its affinity for a K^+^ ion.

To find out which site is more likely to be the binding site for K^+^, we calculate the affinity and the selectivity of the three sites for K^+^, using FEP and TI calculations as described in Methods. The FEP values show up to 3 kcal/mol difference between the forward and backward calculations whereas hysteresis in the TI values remains within 1 kcal/mol. This is because TI allows for longer simulations of fewer windows, which increases sampling and reduces the observed hysteresis. Therefore, we report only the TI results here but stress that very similar results are obtained from the averages of the forward and backward FEP values. The results are summarized in Table 7 and evidence for the convergence of the TI calculations is provided in Figure S5 in File S1. Site 1 has by far the highest affinity of all sites (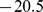 kcal/mol), with a low selectivity margin for Na^+^; the binding free energy decreases by only 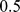 kcal/mol when we go from K^+^ to Na^+^. Site 2 has an affinity of 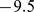 kcal/mol and is highly selective for Na^+^ with a selectivity free energy of 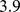 kcal/mol. Site 3 has the lowest affinity (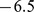 kcal/mol) but this site is highly selective for K^+^, having 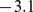 kcal/mol lower binding free energy compared to Na^+^ in agreement with previous valence studies in an EAAT3 model [46].

**Table 7:** Binding free energies for K^+^ and K^+^/Na^+^ selectivity free energies at the three sites proposed for the inward-facing EAAT3.

Ion	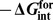	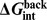	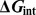	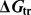	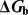
K^+^-site 1	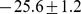	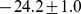	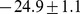	4.4	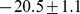
K^+^-site 2	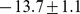	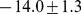	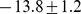	4.3	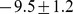
K^+^-site 3	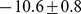	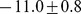	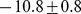	4.3	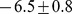
K^+^/Na^+^-site 1	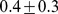	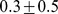	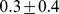	0.2	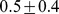
K^+^/Na^+^-site 2	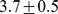	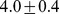	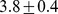	0.1	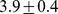
K^+^/Na^+^-site 3	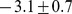	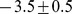	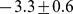	0.2	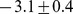

The forward and the backward calculations of the interaction energy are listed in the second and third columns, respectively, and their average in the fourth column. The total binding free energy that includes the translational free energy difference (fifth column) is given in the last column. Errors are estimated from block data analysis using 100 ps windows. All energies are in kcal/mol.

The three K^+^ sites tested here are all based on a model which was initially built from the Glt_Ph_ structure with all the ligands bound. In a recent study, the crystal structure of Glt_Tk_—a close homolog of Glt_Ph_—was resolved in a substrate-free state and in the absence of Na^+^ ions [56]. This structure shows a few conformational changes compared to the holo state of Glt_Ph_, in particular, the region around TM7 and TM8 which is close to our K^+^ site 1. Comparison of this region in Glt_Tk_ and our apo state of EAAT3 shows that such conformational changes are well reproduced in our model (Figure S6 in File S1). We stress that Glt_Tk_, like Glt_Ph_, does not interact with K^+^ ions, so its crystal structure cannot be used directly in search of K^+^ binding sites. For example, the K^+^ binding site 2 is not formed because Glt_Tk_ has a glutamine at the position equivalent to E374 in EAAT3. Similarly, formation of the K^+^ site 3 is compromised in Glt_Tk_ because D398 (D444 in EAAT3) interacts with R401.

The turnover rate of Glt_Ph_ is 0.29 min^−1^ at 30°C [32], and we have suggested in a previous study of Glt_Ph_ that the release of the Na3 ion is the rate-limiting step in the transport cycle due to its high affinity [40]. The speed of transport in EAATs is much higher, with turnover rates of EAAT2 and EAAT3 at room temperature being 15 s^−1^ and 90 s^−1^, respectively. In this case, experimental evidence from EAAT2 and EAAT3 points to the K^+^-dependent relocation step as rate-limiting in the transport cycle [8], [57]. A K^+^ ion at site 1, which is the site with the highest affinity, would be consistent with a model in which this ion would replace the Na^+^ ions during the release of the ligands, therefore making this process faster than in Glt_ph_. This K^+^ ion could again be replaced by Na^+^ ions once the transporter goes back to the outward facing state, restarting the cycle. This last step is consistent with the MD simulations of Glt_Ph_, which show that the first Na^+^ ion to bind to the transporter occupies a transient state, equivalent to the K^+^-site 1, before moving to the Na3 site [41]. It is important to note that this model is based only on the thermodynamics and ion binding, and does not take into account conformational changes of the transporter, which may indicate a different picture. Such conformational changes happen in time scales that are not accessible by all-atom MD simulations, and therefore, are difficult to analyze.

After the completion of this work, two FRET studies were published on the outward to inward transitions of Glt_Ph_ and the time scales involved in them. In one of them, the translocation of the binding pocket from the outward state to the inward state is observed in the order of minutes, suggesting that this transition is the rate-limiting step in the transport cycle [58]. The second study asserts that the transporter is always in a very dynamic state with the transitions between the outward and inward states occurring in the order of seconds regardless of which ligands are bound to it, and furthermore no long dwell times are observed [59]. Interestingly, this last study also shows that Glt_Ph_ cannot perform the full outward to inward translocation (or vice-versa) in the presence of the bound Na^+^ ions—it can only alternate between an intermediate and an inward/outward conformation. The results of Ref. [59] are more consistent with our model, but further experiments and simulations are needed to clarify this issue.

## Conclusions

In this work we have performed MD simulations on two homology models of the mammalian glutamate transporter EAAT3—one in the outward- and the other in the inward-facing conformation—based on the crystal structures of the archaeal homolog Glt_Ph_. We have evaluated the quality of the EAAT3 models using three different methods and shown the reliability of the structures especially in the region of the substrate binding. We have examined the coordination of the ligands in EAAT3, and observed that Glu is stable in the binding site only if the residue E374 is protonated. This is in good agreement with numerous experiments that point to this residue as the proton carrier in EAATs. Regarding the opening of the EC- and IC-gates, our simulations show that the EC-gating in EAAT3 is very similar to that in Glt_Ph_ whereas the IC-gate in EAAT3 exhibits a larger opening compared to Glt_Ph_. This difference can be traced to the change in the position of an arginine residue from the HP1 segment in Glt_Ph_ to the TM8 segment in EAATs, which creates a salt bridge that stabilizes the open state and also helps the release of the Glu substrate to the intracellular media. We have also investigated an apparent disagreement between the crystal structure and the mutagenesis experiments regarding the role of the D455 residue in Na^+^ binding and the possible protonation of this residue. According to our simulations, when D455 is protonated or mutated to an asparagine, the substrate-free EAAT3 cannot support the binding of two Na^+^ ions at the Na1 and Na3 positions. This disagreement can be explained if we assume that only one Na^+^ ion binds to the D455N mutant before the substrate in contrast to two in the wild-type transporter.

We have considered three potential sites for the counter-transported K^+^ ion. Free energy calculations of K^+^ binding at these sites reveal that a site which overlaps with the Na1 and Na3 binding sites has by far the highest affinity of the three. We suggest that binding of K^+^ to this site, which does not happen in Glt_Ph_, could be responsible for the much faster turnover rate in EAAT3 compared to the archaeal transporter. By looking at these results, we can speculate that the proton co-transport and the K^+^ counter-transport might have evolved together and are closely related. As pointed out above, to speed up the transport rate, one needs to exchange the last Na^+^ ion with K^+^ in the inward-facing EAAT3 and counter-transport this K^+^ ion. This extra positive charge in EAAT3 relative to Glt_Ph_ is neutralized by the residue E374 which is a glutamine in Glt_Ph_. In the fully bound, outward-facing EAAT3, protonation of E374 results in the same charge content as in the fully bound, outward-facing Glt_Ph_. Thus the charge content in the binding pocket of Glt_Ph_ is preserved in the EAAT3 transport cycle despite the co- and counter-transport of extra ions. To summarize, proton co-transport via E374 and its subsequent deprotonation enables K^+^ counter-transport. This mechanism might also explain why mutations in the vicinity of the proposed protonation site, like E404D and Y403F in EAAT2 and E374Q in EAAT3, either lock the transporter in the exchange mode by not allowing the counter-transport of K^+^ or significantly reduce this step in the transport cycle (Table 1).

## Methods

### Simulation Details

The simulation system is prepared using the software VMD [60]. We embed the EAAT3 trimer in a 1-palmitoyl-2-oleoyl-sn-glycero-3-phosphocholine (POPC) phospholipid bilayer. Each monomer has four ligands bound: three Na^+^ ions, namely Na1, Na2 and Na3, and Asp as the substrate. We then solvate the protein-membrane complex in a box of water molecules with 48 Na^+^ ions and 45 Cl^−^ ions. The extra Na^+^ ions are required to keep the system neutral, and their number changes depending on the ligands bound to the transporter. For the outward state there are a total of 244 lipid molecules and 15,953 water molecules, and for the inward state 239 lipid molecules and 15,871 water molecules, with a total of 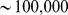 atoms in each simulation box. The two systems are equilibrated in three stages. In the first stage, the coordinates of the ligands and protein atoms are fixed and the system is equilibrated with 1 atm pressure coupling in all directions until the correct water and lipid densities are obtained. At this point, the 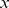 and 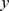-dimensions of the simulation box are fixed, and pressure coupling is applied only in the 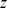 direction thereafter (typical dimensions of the simulation box for both cases are 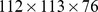 Å^3^). In the second stage, the protein is gradually relaxed by reducing the restraints on the protein and ligand atoms in several steps during MD simulations lasting 2.4 ns. At the end of this stage we are left only with a restraint of 0.1 kcal/mol/Å^2^ in all the backbone atoms of the trimer. Following this, we perform a 2.5 ns alchemical transformation from the Asp substrate to Glu in the three chains, and equilibrate the system with Glu for another 5 ns by gradually releasing the backbone restraints.

All the MD simulations are performed using the NAMD package (version 2.9) [61] with the CHARMM36 force field [62]. The temperature is maintained at 300 K using Langevin damping with a coefficient of 5 ps^−1^, and the pressure is kept at 1 atm using the Langevin piston method with a damping coefficient of 20 ps^−1^ [63]. Periodic boundary conditions with the particle-mesh Ewald method are employed to calculate the electrostatic interactions without truncation. The Lennard-Jones (LJ) interactions are switched off between 10 and 12 Å using a smooth switching function. A time step of 2 fs is used in all MD simulations. Details of all the MD simulations we have performed in the outward and inward models of EAAT3 are summarized in Tables S1 and S2, respectively, giving the conditions and the total running time for each simulation.

### Free Energy Calculations

We perform free energy calculations to determine the standard binding free energy of a K^+^ ion at different sites in the protein, as well as the K^+^/Na^+^ selectivity. For this purpose, we use the free energy perturbation (FEP) and thermodynamic integration (TI) methods [64], as in our previous studies of Na^+^ binding to Glt_Ph_ [40], [42]. We note that the binding free energies calculated using the crystal structures cannot be compared directly to the experimental values due to the large conformational changes occurring in Glt_Ph_ following the binding/unbinding of the Na^+^ ions [65]. Nevertheless they are still a useful tool for finding relative affinities of different sites, as we have done in locating the Na3 site Glt_Ph_ [6].

In the FEP method, the interval between 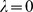 and 1 is divided into 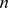 subintervals with 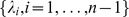, and for each subinterval the free energy difference is calculated from the ensemble average
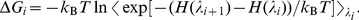
(1)where 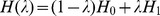, with 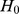 and 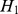 representing the free-ligand and bound-ligand states. The free energy difference is obtained from the sum, 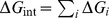. In the TI method, the ensemble average of the derivative, 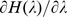, is obtained at several 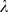 values, and the free energy difference is calculated from the integral:
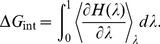
(2)


Provided the integrand can be fitted well with a polynomial, Gaussian quadrature provides an efficient and accurate method for evaluation of such integrals because it allows for longer sampling of a smaller number of windows. The binding free energy of a K^+^ ion is calculated by alchemically transforming a a K^+^ ion to a water molecule in the binding site, while at the same time the opposite transformation is performed in bulk. This approach is justified by the observation that, in the absence of a K^+^ ion, a water molecule always occupies the binding site. For the Na^+^/K^+^ selectivity, we replace a K^+^ ion with a Na^+^ ion in the binding site, and vice-versa in bulk. In the FEP simulations we use 66 exponentially spaced windows with 20 ps of equilibration plus 40 ps of production for each window. In the TI method, we use seven windows starting from the FEP runs, and run 0.4 ns of equilibration plus 0.8 ns of data collection for each window. These parameters have been found to be optimal in previous studies of Glt_Ph_ [40], [42]. We report here only the TI results because they show better convergence, but we always check for consistency between the two methods, as well as performing both the forward and backward transformations in each case. When calculating the standard binding free energies, we also have to account for the loss of translational entropy of the ligand upon binding. The translational free energy difference is determined using [66], [67]

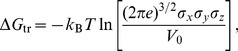
(3)where 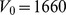 Å^3^, 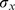, 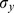, and 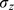, are the principal rms fluctuations of the center of mass of the ligand in the binding site, estimated from 5 ns of MD simulations of the bound ion with no restraints applied. The standard binding free energy of the ion can then be expressed as [68], [69]:
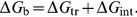
(4)


In this approach, the force field contribution to the free energy difference between the bound and unbound states (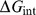) is calculated using the alchemical FEP and TI methods, with a restrained ion/water molecule in the binding site/bulk solvent. To make sure that these restraints have little effect on 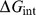, values of the spring constant are determined from the expression 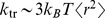, where 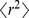 is the mean-square fluctuations of the unrestrained ion in the binding site, which gives 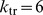 kcal/mol/Å^2^. The same goes for the replacement of a water molecule, which also samples a small volume in the ion binding site. In the bulk solvent, the restraints have no effect in the force field contribution to the free energy because bulk water is an isotropic medium. The free energy change due to loss of the translational entropy upon binding of the ion (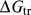) is calculated separately using Eq. 3, where no restraints are employed. A more detailed validation of the method is given in a recent study of ligand binding to the ionotropic glutamate receptor GluA2 [70]. We note that the alchemical transformation approach adapted here is independent of path, and differs from the path-dependent methods that use a physical reaction coordinate. In the latter case, the ligand needs to be restrained within a cylinder or a cone while moving along the reaction coordinate, and this restriction has to be taken into account in calculating the binding affinity at the standard concentration [71], [72].

## Supporting Information

File S1 **Supporting Figures and Tables. Figure S1, Snapshots of the outward and inward-facing models of EAAT3 including the TM 4B-4C loop. Figure S2, DOPE energy profiles for the outward and inward-facing models of EAAT3. Figure S3, Ramachandran plots for the templates and models used in this study. Figure S4, Backbone RMSDs for the outward and inward-facing models of EAAT3. Figure S5, Convergence of the TI results for the binding free energies of K^+^ ion. Figure S6, Comparison of the Glt_Tk_ crystal structure with the apo state of the EAAT3 model. Table S1, Summary of the MD simulations performed for the outward-facing model of EAAT3 in this study. Table S2, Summary of the MD simulations performed for the inward-facing model of EAAT3 in this study.**
(PDF)

File S2 **Movie showing the release of the Asp substrate to the solvent in the Q318E mutant of Glt_Ph_.**
(MPG)
