# Supplementary material for: Molecular Dynamics Simulations of the Mammalian Glutamate Transporter EAAT3
Source: PLoS One. 2014 Mar 18;9(3):e92089. doi: 10.1371/journal.pone.0092089 (PMC3958442; doi:10.1371/journal.pone.0092089)
Supplement: File S1 — Supporting Figures and Tables. Figure S1, Snapshots of the outward and inward-facing models of EAAT3 including the TM 4B-4C loop. Figure S2, DOPE energy profiles for the outward and inward-facing models of EAAT3. Figure S3, Ramachandran plots for the templates and models used in this study. Figure S4, Backbone RMSDs for the outward and inward-facing models of EAAT3. Figure S5, Convergence of the TI results for the binding free energies of K+ ion. Figure S6, Comparison of the GltTk crystal structure with the apo state of the EAAT3 model. Table S1, Summary of the MD simulations performed for the outward-facing model of EAAT3 in this study. Table S2, Summary of the MD simulations performed for the inward-facing model of EAAT3 in this study. (PDF) [file pone.0092089.s001.pdf]

Supporting Information for
Molecular Dynamics Simulations of the Mammalian Glutamate Transporter EAAT3

Germano Heinzelmann and Serdar Kuyucak

School of Physics, University of Sydney, NSW 2006, Australia

Table 1: Equilibrium simulations performed in this study for the outward-facing model of EAAT3.

Mut.	Time	Glu	Na1	Na2	Na3	Comments
E374p	60ns	+	+	+	+	Transporter remained stable with gate closed, Na2 was released in two of the chains.
None	20ns	+	+	+	+	Instability in the substrate and in the HP2 gate, Na2 was released in all of the chains.
None	60ns	-	+	-	+	Opening and large fluctuations of the HP2 gate in all of the chains, Na^+ ions remained bound.
None	60ns	-	-	-	-	Opening and large fluctuations of the HP2 gate observed in all of the chains.
D455N	10ns	-	+	-	+	Na1 is released back to the solvent in two of the chains of the transporter.
D455N	10ns	-	+	-	-	Na1 remains bound coordinated by D368 in all of the chains of the transporter.
D455p	10ns	-	+	-	+	Na1 is released back to the solvent in all of the chains of the transporter.
D455p	10ns	-	+	-	-	Na1 remains bound coordinated by D368 in all of the chains of the transporter.

Table 2: Equilibrium and FEP simulations performed in this study for the inward-facing model of EAAT3.

Mut.	Time	Glu	Na1	Na2	Na3	K^+	Comments
E374p	60ns	+	+	+	+	-	Transporter remained stable with gate closed, Na2 was released in one of the chains.
None	20ns	+	+	+	+	-	Instability in the substrate and in the HP1-HP2 gate, Na2 was released in all chains.
None	60ns	-	+	-	+	-	Opening of the HP1-HP2 gates in all of the chains, Na^+ ions remained bound.
None	60ns	-	-	-	-	-	Opening of the HP1-HP2 gates in all of the chains, with a large displacement of HP1.
None	50ns	-	-	-	-	s-1	K^+ remains bound at site 1 during the whole simulation.
None	50ns	-	-	-	-	s-2	K^+ remains bound at site 2 in two of the chains during the whole simulation.
None	50ns	-	-	-	-	s-3	K^+ remains bound at site 3 during the whole simulation.
FEP simulations							
None	16.8ns	-	-	-	-	s-1	K^+ binding affinity of -20.5 kcal/mol at site 1.
None	16.8ns	-	-	-	-	s-1	K^+ selectivity of 0.5 kcal/mol at site 1.
None	16.8ns	-	-	-	-	s-2	K^+ binding affinity of -9.5 kcal/mol at site 2.
None	16.8ns	-	-	-	-	s-2	K^+ selectivity of 3.9 kcal/mol at site 2.
None	16.8ns	-	-	-	-	s-3	K^+ binding affinity of -6.5 kcal/mol at site 3.
None	16.8ns	-	-	-	-	s-3	K^+ selectivity of -3.1 kcal/mol at site 3.

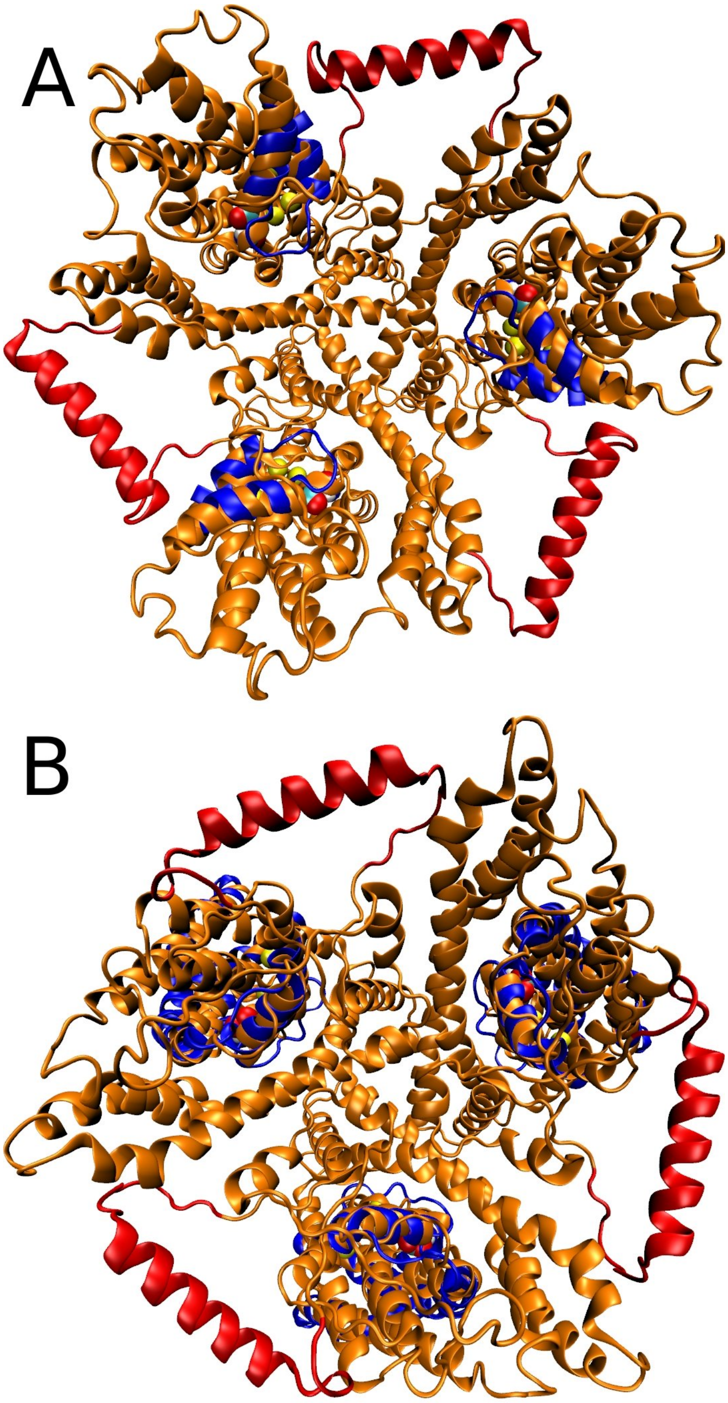

Figure 1: (A) The model for the outward-facing state of EAAT3 including the 4B-4C loop 50-residue segment—which is not present in our model–placed at the position proposed in Ref. 51 (shown in red). The position of the HP2 segment in the open state, which is the segment that goes through conformational changes during the opening, is shown in blue. The substrate and sodium ions in the binding site are also shown. (B) The same for the inward-facing model of EAAT3, showing the segments that undergo conformational changes during the inward opening in blue, namely, HP1, HP2 and TM8. In both cases, the bound ligands and the conformational changes that happen during the opening of EAAT3 are not in proximity to the 4B-4C loop.

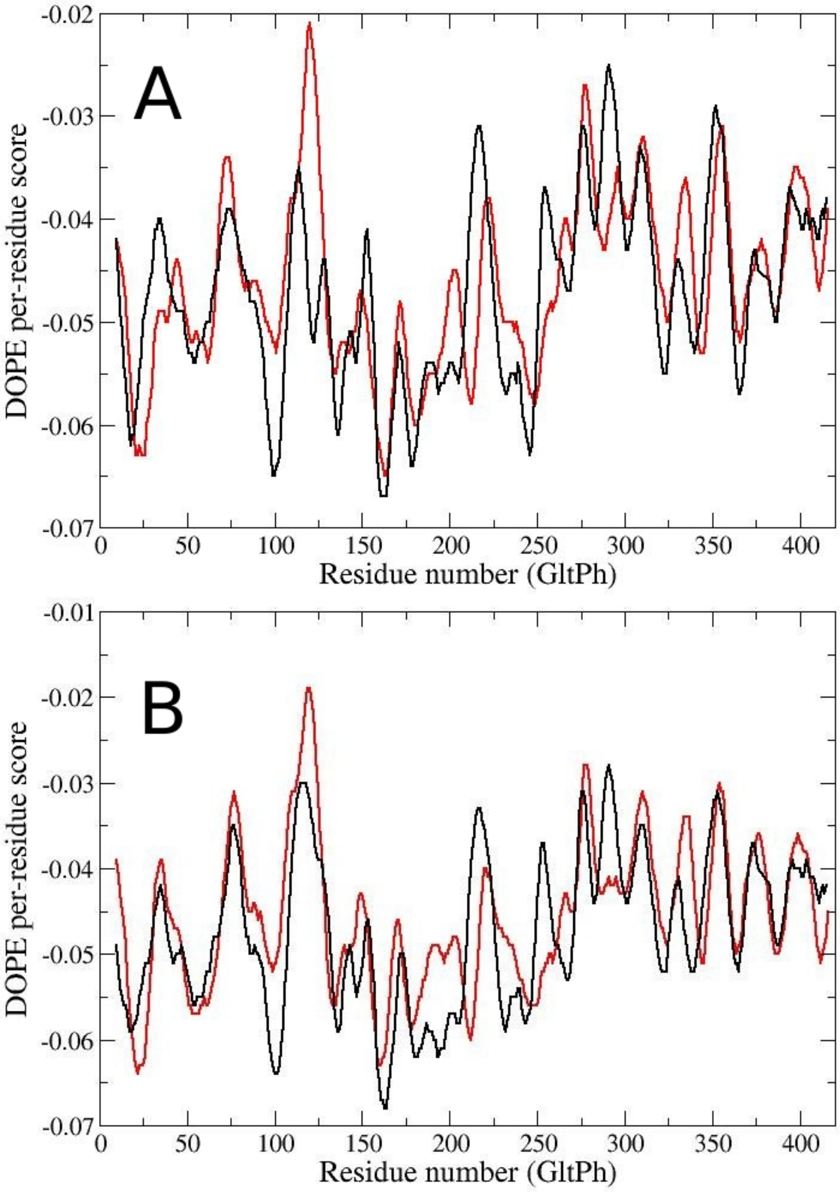

Figure 2: (A) DOPE per-residue score for the 2NWX crystal structure (red) and the EAAT3 model in the outward-facing state (black). The residues are numbered according to the $\mathrm{Glt_{Ph}}$ sequence. (B) The same for the 3KBC crystal structure (red) and the EAAT3 model in the inward-facing state (black).

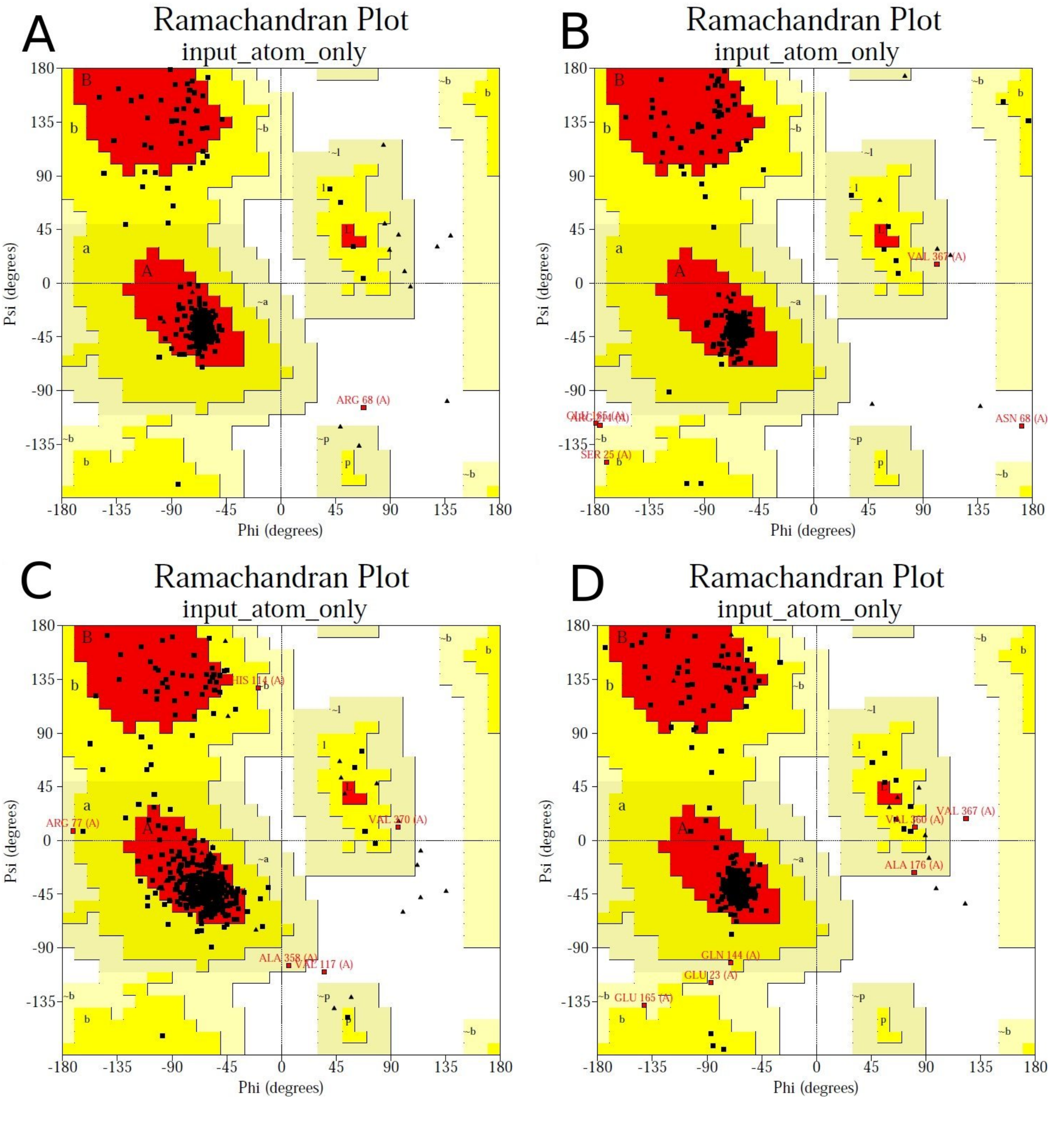

Figure 3: Ramachandran plots for the templates and models used in this study.(A) 2NWX crystal structure.(B) EAAT3 model in the outward-facing conformation.(C) 3KBC crystal structure.(D) EAAT3 model in the inward-facing conformation.

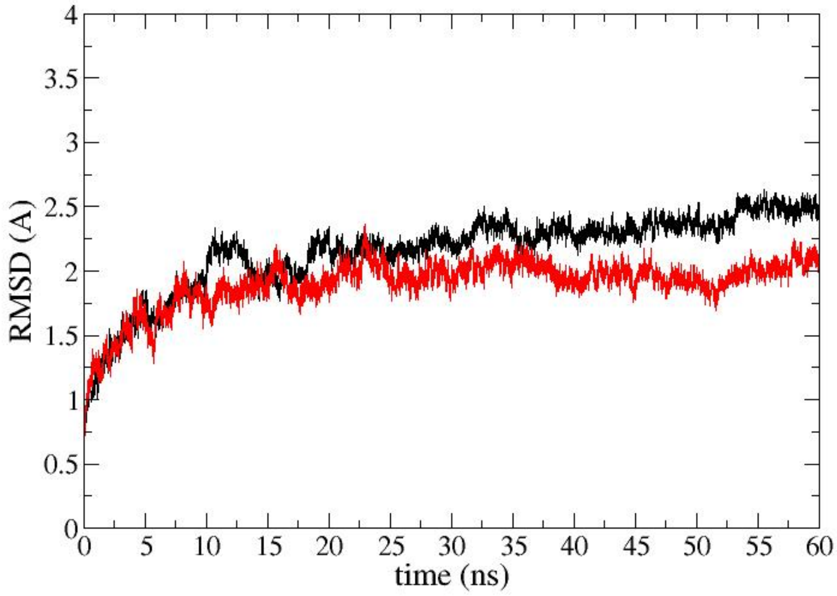

Figure 4: RMSD of the protein backbone during 60 ns of simulations with all ligands bound and the protonated E374 side-chain, in the outward (black) and inward (red) states of EAAT3. The system shows no large conformational changes after 10 ns, which indicates that the models are stable.

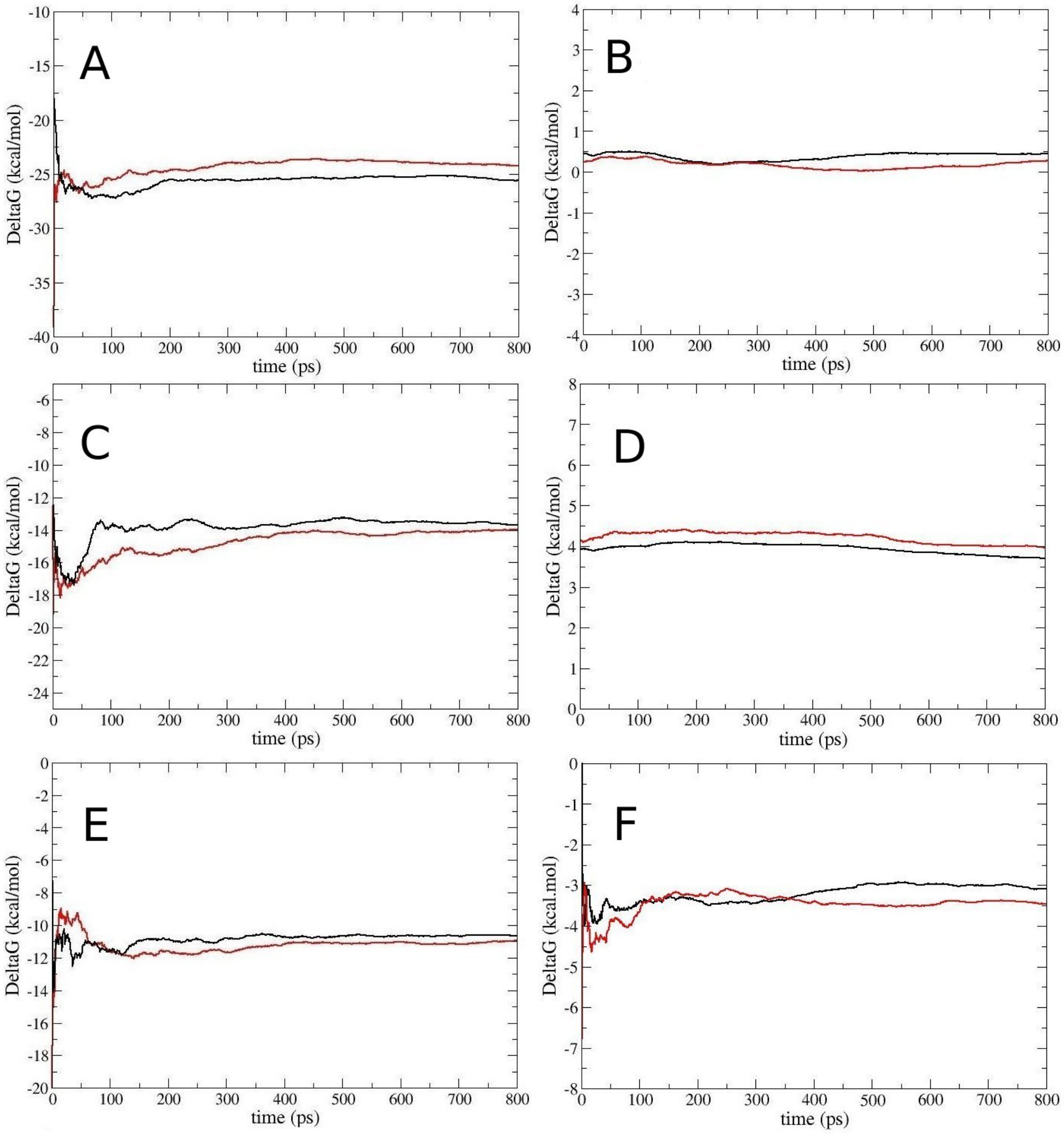

Figure 5: Convergence of the TI results in the calculation of the binding free energies of K^+, and also the K^+/Na^+ selectivity. In all graphs the negative of the forward and the backward transformations results are shown with black and red lines, respectively. (A) Interaction free energy and (B) selectivity of the K^+ ion at site 1. (C,D) The same for the site 2. (E,F) The same for site 3.

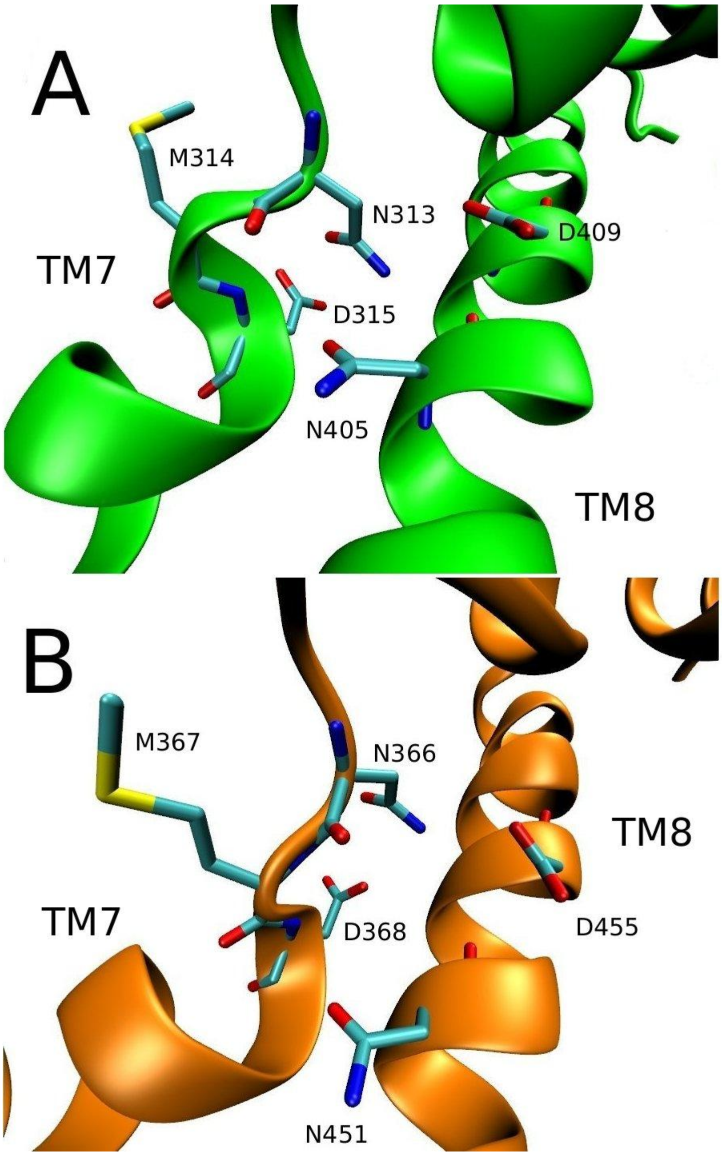

Figure 6: Comparison of the conformations around the TM7–TM8 region in the Glt_{Tk} crystal structure (A) and in our simulations of the apo state of EAAT3 (B). Conformational changes that are observed between the loaded and apo states of the crystal structures are captured in our apo state model of EAAT3. Only the functionally important residues in this region are indicated explicitly.
